# COVID-19 Mortality Rate Prediction for India Using Statistical Neural Network Models

**DOI:** 10.3389/fpubh.2020.00441

**Published:** 2020-08-28

**Authors:** S Dhamodharavadhani, R Rathipriya, Jyotir Moy Chatterjee

**Affiliations:** ^1^Department of Computer Science, Periyar University, Salem, India; ^2^Department of IT, Lord Buddha Education Foundation (LBEF), Kathmandu, Nepal

**Keywords:** Covid-19, mortality rate prediction (MRP), statistical neural network (SNN), probabilistic neural network (PNN), generalized regression neural network (GRNN), radial basis function neural network (RBFNN), non-linear autoregressive (NAR), root mean square error (RMSE)

## Abstract

The primary aim of this study is to investigate suitable Statistical Neural Network (SNN) models and their hybrid version for COVID-19 mortality prediction in Indian populations and is to estimate the future COVID-19 death cases for India. SNN models such as Probabilistic Neural Network (PNN), Radial Basis Function Neural Network (RBFNN), and Generalized Regression Neural Network (GRNN) are applied to develop the COVID-19 Mortality Rate Prediction (MRP) model for India. For this purpose, we have used two datasets as D1 and D2. The performances of these models are evaluated using Root Mean Square Error (RMSE) and “R,” a correlation value between actual and predicted value. To improve prediction accuracy, the new hybrid models have been constructed by combining SNN models and the Non-linear Autoregressive Neural Network (NAR-NN). This is to predict the future error of the SNN models, which adds to the predicted value of these models for getting better MRP value. The results showed that the PNN and RBFNN-based MRP model performed better than the other models for COVID-19 datasets D2 and D1, respectively.

## Introduction

At the end of December 2019 in Wuhan, China, it was first reported that a human infection was caused by a novel coronavirus (nCov) or Wuhan virus or 2019-nCov (1). One of the biggest challenges of this epidemic is the human-to-human transition of nCov. The coronavirus (COVID-19) infected cases increase at an exponential rate worldwide. On 30 January 2020, the World Health Organization (WHO) issued a worldwide health emergency warning notice (2), labeling that 2019-nCoV is of urgent global concern. The disease and mortality rates for the COVID-19 are uncertain at the early stage (3) especially for young ones and aged people. WHO has estimated the reproduction factor (R0) of nCov is 2.7. In demand to control the extensive and quick spread of the nCov, public health sectors took reliable preventative measures and imposed curfew or lockdown infested cities in China, United States, India, and other countries also (4, 5). This is to limit the social distance between people and to avoid the broadcast of this novel virus via humans to humans.

Since 2000, machine learning techniques have gain momentum and play a vital role in epidemiological data analysis. Machine learning techniques also can be used to develop standard mortality models. Deprez et al. (6) used machine learning algorithms to fit and assess the mortality model by detecting the weaknesses of different mortality models. Artificial Neural Networks (ANNs) (7) used to track and forecast latent mortality factors with greater predictability. Richman and Wüthrich (8) have used the Lee-Carter model to many population predictions using neural networks.

“Probabilistic Neural Network (PNN)” is used for kernel analysis. PNN makes training faster (9). PNN assimilates statistical concepts with neural networks and thus outcomes in an adjusting classification system in which conventional statistical equivalents have unsuccessful. The PNN used to describe bacterial growth and no growth states and to assess the probability. Evolution as affected by changing working conditions (10).

In (11), the GRNN model was created as another possible instrument for the infectious disease rate expectation field. Han et al. (12) built a GRNN network with one-dimensional input and output layer to forecast the occurrence of blood and sexually transmitted diseases. Hong and Zhou (13) made a comparison study on back propagation neural network (BPNN), GRNN, and RBFNN for evaporation prediction. The results revealed that PNN is a powerful technique than artificial neural network methods.

Montazer et al. (14) carried out a large scale comparison study for the major machine learning models such as multilayer perceptron, Bayesian neural networks, RBFNN, GRNN (also called kernel regression), K-nearest neighbor regression, CART regression trees, support vector regression, and Gaussian processes for time series forecasting. The authors reported that these models have different impacts on the performance purely dependent on the dataset.

The RBF and GRNN (15) have been applied over heart disease patient data for the outcome of the medicine. The results showed that RBF performed well for prescribing medicine for the patient. The RBF and GRNN have been applied over heart disease patient data for the outcome of the medicine (16). The results showed that RBF performed well for prescribing medicine for the patient. Hajmeer and Basheer (17) claimed that the Gaussian process approach performed better than the standard generalized linear model (GLM) for the Phenomenological forecasting of dengue disease incidence. Huber (18) reviewed various learning methods for defining network parameters such as widths, centers, and synaptic weights of the RBF neural network. Williams and Rasmussen (19) general regression neural networks for forecasting time series data was proposed as an automated methodology. This methodology is meant to achieve an effective and fast tool so that a huge amount of time series can be predicted automatically. From these works, one could clearly understand the applications of PNN, GRNN, and RBFNN in various research domains.

In recent years, Artificial neural networks (ANNs) have been used frequently to capture the uncertainty in the time series dataset as they have been proven to be a powerful technique for handling the non-linear data (13). Therefore, the use of these ANN techniques gains huge momentum in recent years in the field of epidemiological predictions for the linear, non-linear, and hybrid data (20–22). Hybrid technique integrating the Autoregressive Integrated Moving Average (ARIMA) with a Non-linear Auto-regressive Neural Network (NAR) yielded better forecasting accuracy for time series data (20) relative to other combinations of ANN models or time series models individually (21) proposed the SARIMA-NARX technique for the prediction of scarlet fever incidence cases in China. Moreover, the authors claimed that this hybrid technique has the promising ability to handle both linearity and non-linearity in the scarlet fever dataset than the other techniques. Wang et al. (21) developed techniques by fusing a seasonal autoregressive integrated moving average (SARIMA) with a neural network non-linear autoregression (NNNAR) for tuberculosis (TB) incidence data in china.

Singh et al. (22) used an advanced ARIMA model for predicting the COVID-19 disease spread using Top 15 COVID-19 affected countries. They forecasted that the recovery and death rates were rose faster in the next 2 months when compared to COVID-19 confirmed cases (23). A fine-tuned Random Forest model was proposed by Iwendi et al. (24) for prediction of the severity of the COVID-19 case using the migration, geographical, demographic, and travel details of COVID-19 patients. Tomar and Gupta (25) used Long Short-Term Memory (LSTM) and curve fitting for forecasting the number of COVID-19 confirmed cases in India 30 days ahead. The main limitation is that the proposed method is accurate only for a short range of values (26).

In accordance with the relevant literature, the error variable has not been considered in the modeling of standard neural network models and hybrid neural network models for the improvisation of epidemiological prediction accuracy. Therefore, this work aimed to propose Statistical Neural Network models and their hybrid version (PNN, GRNN, and RBFNN) with a NAR model, to predict COVID-19 mortality rate prediction in India by considering the error variable. Moreover, to evaluate the performance of these models, a benchmark measure, the RMSE, is used. The results of this study may facilitate the public health officials of the Indian government for better prevention and control measures for COVID-19.

The remaining part of this article is arranged as follows. Section Methods and Materials explains about the methods and materials for forecasting of COVID-19 Mortality for India. Section Proposed Methodology expounds on the proposed methodology for COVID-19 Death case prediction. Section Result and Discussion discusses about the results of this study. Section Conclusion and Future work summarizes this work with possible future work.

## Methods and Materials

### Dataset Description

For experimentation purpose we have used (27) for predicting the Covid-19 death cases for India. This dataset contains India's COVID-19 Confirmed cases and Death cases from January 20, 2020, to May 30, 2020, which is used for training and testing models. First, these data are pre-processed to eliminate missing values and inappropriate values. These data can be used to create two types of datasets. They are:

- Dataset1 (D1) contains a time series of COVID-19 death cases.
- Dataset2 (D2) contains two attributes such as COVID-19 confirmed cases and death cases. Here, “death case” is a predictive attribute and “confirmed case” is a response attributes or independent attribute.

### Probabilistic Neural Network (PNN)

It's a kind of radial basis networks (9). This applies to the Bayesian decision rule and Parzen (estimators of the probability density function), called the Bayes-Parzen classification. PNN contains equally statistical pattern recognition characteristics and BPNN. It applies to various fields including pattern recognition, non-linear mapping, and classification. Equation (2) represents the PNN is a supervised feed-forward neural network. This is similarly made of three layers with an algorithm for one-pass training (10). PNN has the capacity of Train on a sparse collection of data. It's also capable of classifying data to different types of outputs (11). There is plenty of usage of PNN aimed at classification advantages. For instance: The PNN processing time is quicker than BPNN and Robust and noisy. The PNN manner of training is Simple and Immediate (15, 28–31).

(1) $$\begin{array}{r} P\;\left(X_{new}\;|\;C_{i}\right)\;=\;Pi\;=\;\frac{1}{\left|C_{i}\right|}\;\sum_{\;j=1}^{{|C}_{i}|}w_{i,j} \end{array}$$

Where P denotes as probability, X as predicted value, w represents as the weight value, C represents as class, where i indexes the input dimension and w_ji_ is a positive parameter signifying the ith weight of the jth hidden unit.

### Generalized Regression Neural Network (GRNN)

It's a special case of Radial Basis Networks (RBN) (9). The structure of a GRNN is comparatively easy and fixed with 2 layers. The first layer is the pattern and the second layer are the summation. If each unit in the pattern layer is passed through the input, the input-response relationship will be “memorized” and stored in the unit. As a result, in the training set, no. of units in the pattern layer is equal to the no. of actual values. A Gaussian PDF will be added to the network input in each pattern unit, so that represented as the Equation (2)

(2) $$\begin{array}{r} \theta =EXP\left[-0.5{\;}^{*}\;\left(X\;-\;u\right)\;`\left(X-u\right)/\left(\;\sigma \;ˆ\;2\right)\right] \end{array}$$

where **θ** is the Pattern Unit output, X is the input, u is training vector stored in the unit, and σ is a positive constant known as “spread” or “smooth parameter.” If **θ** is calculated, computation is moved on to the summation layer

(3) $$\begin{array}{r} Y|X=SUM\left(Y*\theta \right)\;/\;SUM\left(\theta \right) \end{array}$$

where Y|X is the prediction conditional on X and Y is the response in the training sample (12, 31–39).

### Radial Basis Function Neural Network (RBFNN)

It's a ANN (14, 31, 33) that uses functions on a radial basis as activation functions shown in Equation (5). The RBFNN is a neural network with three layers of feed-forwards. The first layer is linear and only the input signal is transmitted, while the next layer is non-linear and uses Gaussian functions (9, 10). The third layer incorporates the Gaussian outputs in linear form. During training, only the tap weights among the hidden layer and the output layer are changed (30–39).

(4) $$\begin{array}{r} f\left(x\right)=\frac{1}{\sqrt[{\sigma }]{2\pi }}e^{-\frac{{\left(x-\mu \right)}^{2}}{2\sigma^{2}}} \end{array}$$

The function approximation f(x) is a Gaussian function. x represents as the actual values. The input x, to find the dimensional parameters of the function.

### Non-linear Autoregressive Neural Network (NAR-NN)

The NAR (34) is a sort of ANN fitting for evaluating future estimations of the input variable (9, 10). The NAR-NN empowers the forecast of future estimations of a time series. It upheld by its history foundation utilizing a re-feeding care of instrument, in which an anticipated worth may fill in as a contribution for new expectations at further developed focuses in time. In condition (6) speaks to as anticipate arrangement y(t) given d past estimations of y(t).

(5) $$\begin{array}{r} y\left(t\right)=f\left(y\left(t-1\right),\ldots ,y\left(t-d\right)\right) \end{array}$$

Where y represents as input parameter, t denotes as time period and d represents as delay.

### Root Mean Square Error (RMSE)

RMSE (34, 35) is the square root of the square differences measured between predicted and actual COVID-19 Death cases. Its representation is shown in Equation (6).

(6) $$\begin{array}{r} RMSE=\sqrt{\frac{1}{n}\overset{n}{\underset{i=1}{\sum }}{\left({Predicted\;COVID-19\;Death\;Case}_{i}-{Actual\;COVID-19\;Death\;Case}_{i}\right)}^{2}} \end{array}$$

Where *n* = number of samples.

### Correlation Coefficient (R)

It's a measure a linear relationship between the predicted and actual COVID-19 death cases. It represents as in Equation (7)

(7) $$\begin{array}{r} R=\frac{\sum_{i=1}^{n}\left(t_{i}-\bar{t_{l}}\right)\left(p_{i}-\bar{p_{l}}\right)}{\sqrt{\sum_{i=1}^{n}{\left(t_{i}-\bar{t_{l}}\right)}^{2}\sqrt{\sum_{i=1}^{n}{\left(p_{i}-\bar{p_{l}}\right)}^{2}}\;}\;} \end{array}$$

where t is the actual COVID-19 death case value, p is the predicted COVID-19 death case value, $\bar{t}$ is the mean of actual COVID-19 death case value $\bar{p}$ is the mean predicted COVID-19 death cases value, and n is the total number of data points.

## Proposed Methodology

**Figure 1:**
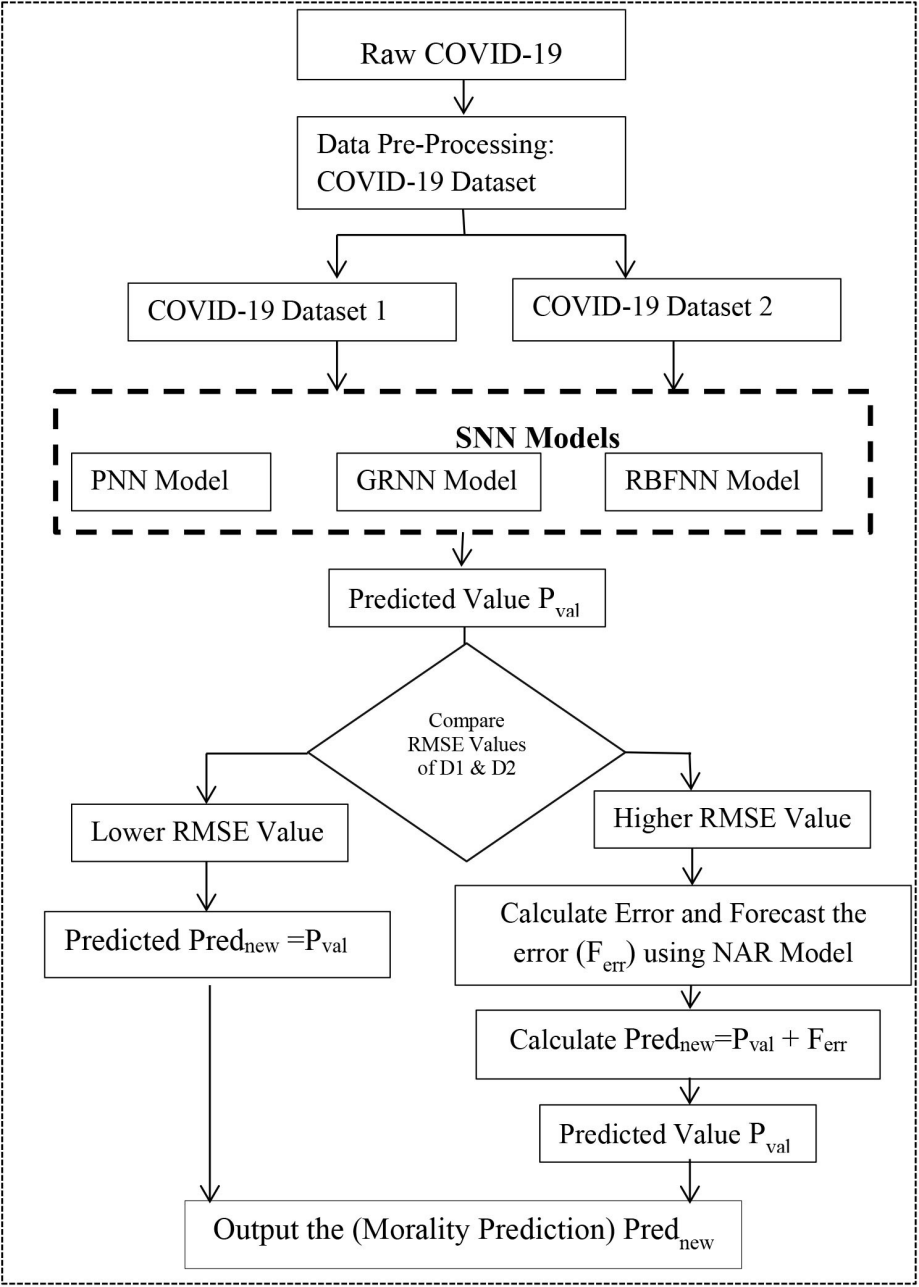
Proposed methodology for COVID-19 MRP model.

**Figure 2:**
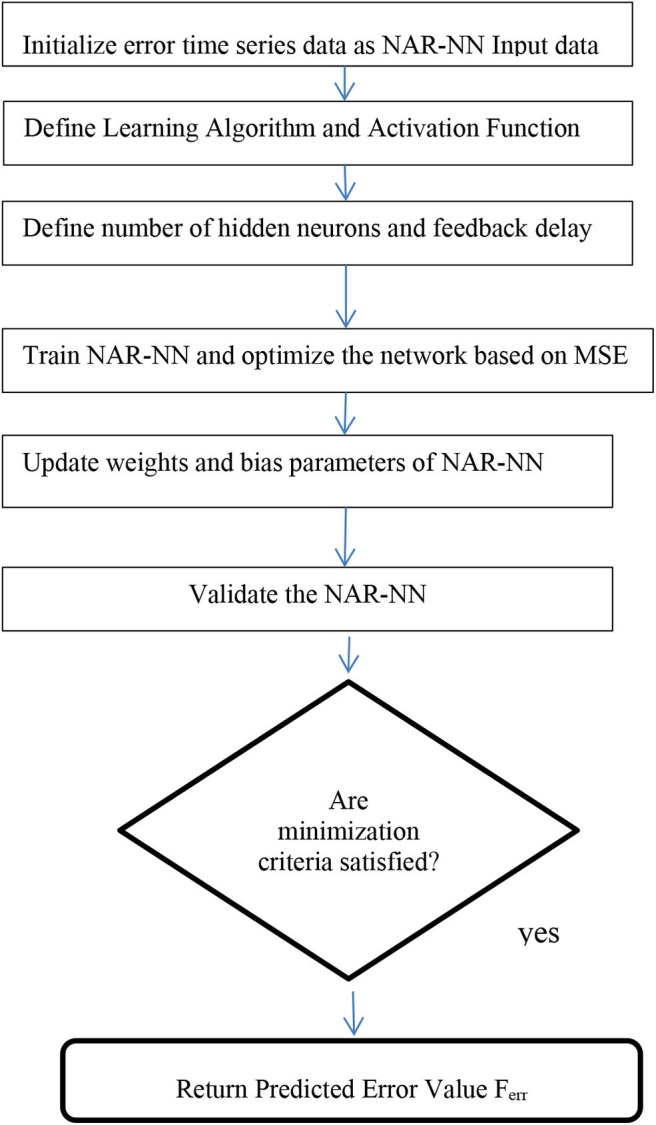
Workflow of NAR-NN time series forecasting.

In this paper, three SNN models (such as PNN, GRNN, and RBFNN) are constructed with the appropriate model parameter values and used in these two datasets to validate the predicted results concerning given the available datasets. Figure 1 illustrates the proposed methodology. The following steps are used to develop the proposed methodology:

Step 1: Pre-process the raw COVID-19 time series dataset. Create (D1) and (D2).
Step 2: Initialize Model Parameters for PNN, GRNN, and RBFNN. Parameters are shown in Table 1.
Step 3: Input D1 and D2 into the PNN model, GRNN model, and RBFNN model, respectively, and predict COVID-19 death cases (Prednew) for “n” period ahead or for given set of confirmed cases.
Step 4: Compare the SNN models of two datasets using RMSE value. Calculate the error or residual of the SNN model with higher RMSE.
Step 5: Input these residuals into the NAR-NN time series forecasting model and predict the residual values (Ferr). It is shown graphically in Figure 2.
Step 6: Ferr is added with PredNew to generate an optimized prediction value.
Step 7: Return optimized predicted values as output.

**Table 1:** Model parameters setup.

**Model parameters**	**PNN model**	**GRNN model**	**RBFNN model**
Hidden layer (HL)	Fixed architecture	Fixed architecture	Fixed architecture
Number of neurons in HL	10–15	10–15	10–15
Training algorithm	Bayesian regularization	Bayesian regularization	Bayesian regularization
SPREAD (σ)	0–4	0–4	0–4
Performance indicator Measure	RMSE	RMSE	RMSE

Figure 2 describes the working principle of the NAR-NN model for error forecasting for these models.

## Result and Discussion

In this section, the results of three different SNNs: PNN, GRNN, and RBFNN models for D1 and D2 are presented and discussed. The performance of these models was compared. The benchmark key performance indicator metrics such as RMSE and Correlation coefficient (R) is used to estimate the COVID-19 Mortality models for India.

In general, residues or errors are an inevitable part of any predictive or regression models. Similarly, there are errors in the PNN, GRNN, and RBFNN models. To provide a predictive model with high accuracy, this study explores a hybrid approach, including the NAR-NN time series forecasting model. For hybridization, first is to find out the mean RMSE value of SNN models for D1 and D2. And, then identify which set of SNN models has the highest mean RMSE value. Here, the mean RMSE value of SNN models for D1 is higher than that of D2. Therefore, trends in residues or errors are detected for D1 and predicted by the NAR-NN model. Combining the predicted residual values with predicted COVID-19 death cases of respective SNN models of D1 for higher predictive accuracy.

Table 2 shows the values of the performance metrics such as RMSE and R2 for three SNN models.

**Table 2:** Performance metrics for datasets.

**Model**	**RMSE**		**R**	
	**D1**	**D2**	**D1**	**D2**
PNN	8.889595	7.898071	0.999978	0.999983
GRNN	9.713768	8.388667	0.999975	0.999981
RBFNN	8.528095	9.50462	0.99998	0.999977

**Figure 3:**
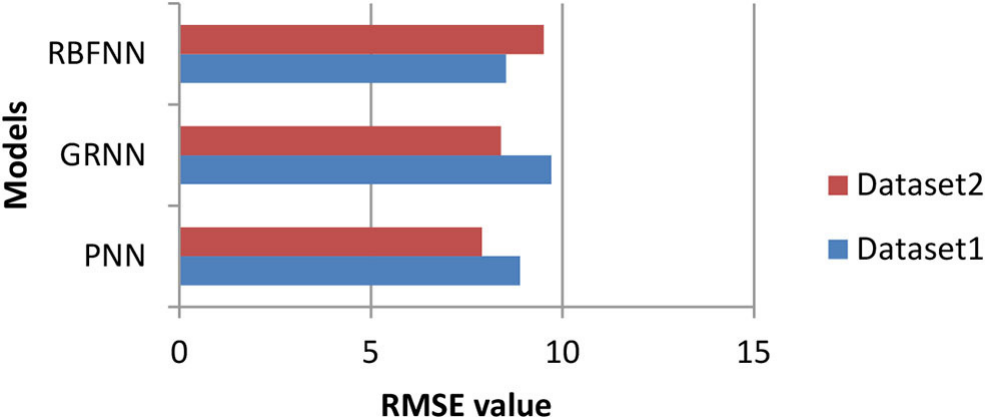
Comparison of RMSE values with a different model.

The performance of these models is compared based on RMSE value as shown in Figure 3. While comparing the mean RMSE value of the three SNN models for COVID19 time series, i.e., D1 data is higher compared to the mean RMSE value of the three SNN models for D2. Therefore, to reduce the RMSE value for D1, the NAR-NN is combined with the SNN models. The purpose of this NAR-NN is to forecast the error of SNN models. Thereafter, this predicted error is included in the predicted COVID19 mortality cases of the respective SNN models for D1.

Table 3 shows the optimum SPREAD values or smoothing factor (σ) of three SNN models. This spread parameter of the SNN models has an important inspiration on the prediction performance. Consequently, in instruction to select the appropriate SPREAD parameter of these models, we run these models with different SPREAD values from 0 to 4 with 0.02 intervals and identified the best SPREAD values of the respective models.

**Table 3:** SPREAD value for PNN, GRNN, and RBFNN.

**Model**	**D1 (spread)**	**D2 (spread)**
PNN	0.5	2
GRNN	4	4
RBFNN	1.5	1.68

**Figure 4:**
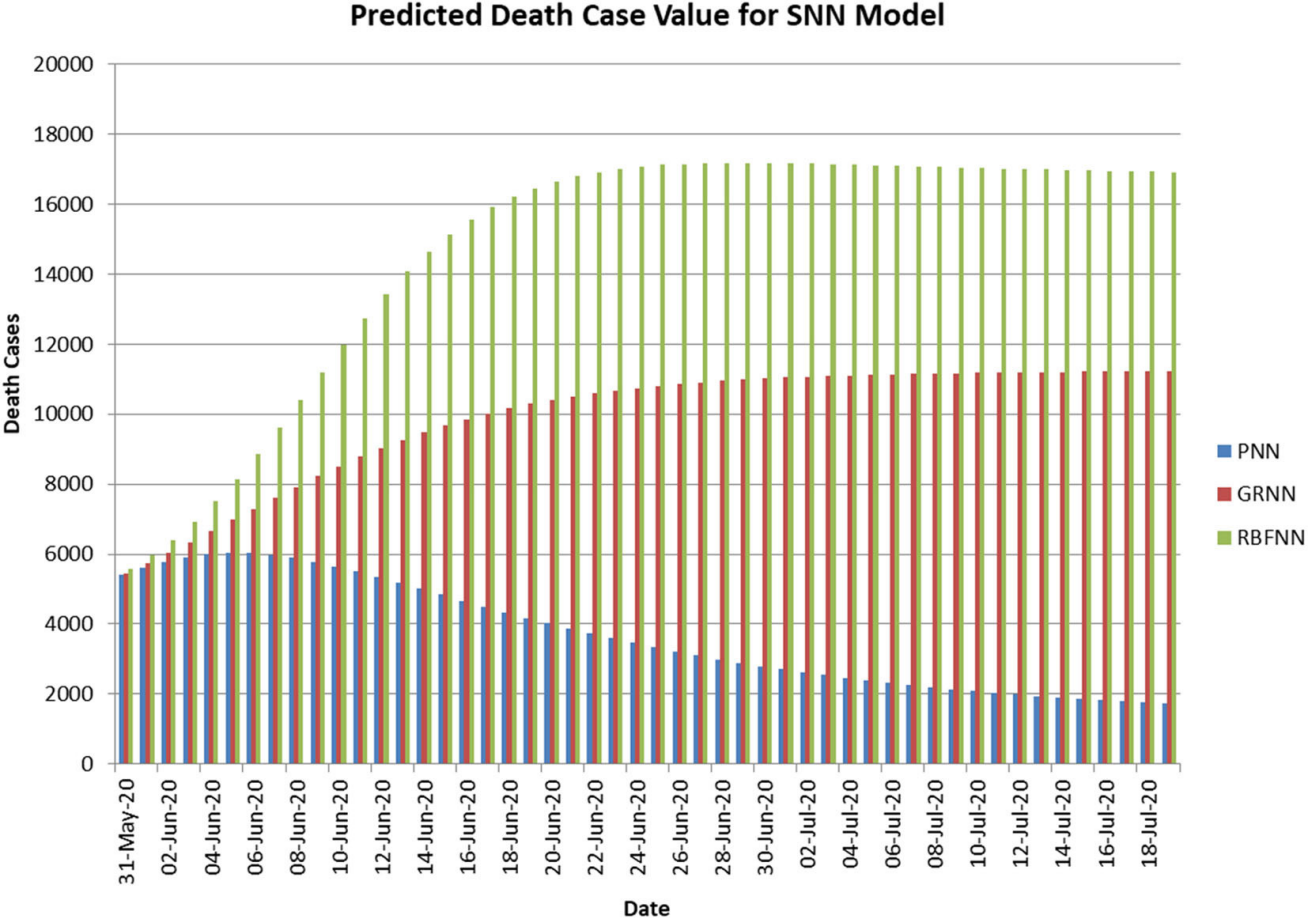
Predicted curve for standard SNN.

**Figure 5:**
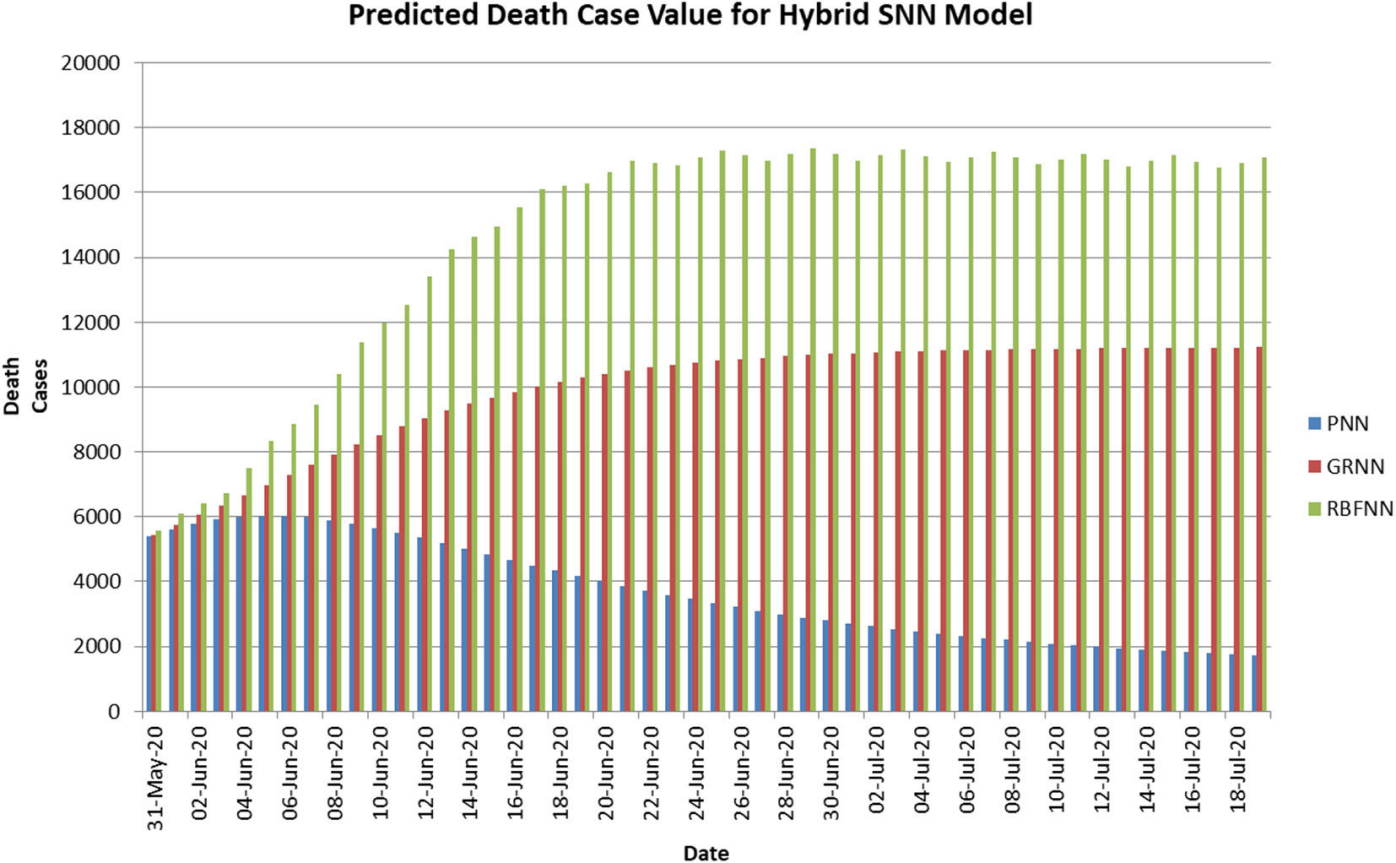
Predicted curve for hybrid SNN.

Figure 4 shows the predicted curve of standard SNN model for COVID-19 death cases, respectively. Here, the X-axis represents the dates and Y-axis represents the number of death cases predicted. Figure 5 shows the predicted curve of hybrid SNN model for COVID-19 death cases, respectively. Here, the X-axis represents the dates and Y-axis represents the number of death cases predicted.

Tables 4, 5 show the predicted number of COVID-19 death cases using time series data (i.e., D1) for three standard and hybrid SNN models. The hybrid model is the combination of standard models and error forecasting model using NAR-NN. There is no difference in the predicted values for the standard and hybrid models since their RMSE value is about 0.2 approximately.

**Table 4:** Predicted value Ypred for D1 using standard models.

**Date**	**PNN**	**GRNN**	**RBFNN**
31-May-20	5,409	5,454	5,574
1-Jun-20	5,608	5,732	5,957
2-Jun-20	5,776	6,026	6,403
3-Jun-20	5,906	6,333	6,917
4-Jun-20	5,990	6,649	7,501
5-Jun-20	6,028	6,970	8,151
6-Jun-20	6,022	7,291	8,859
7-Jun-20	5,976	7,609	9,614
8-Jun-20	5,896	7,920	10,398
9-Jun-20	5,787	8,219	11,191
10-Jun-20	5,656	8,506	11,972
11-Jun-20	5,509	8,776	12,721
12-Jun-20	5,350	9,029	13,422
13-Jun-20	5,183	9,263	14,061
14-Jun-20	5,013	9,479	14,631
15-Jun-20	4,841	9,675	15,129
16-Jun-20	4,670	9,854	15,555
17-Jun-20	4,502	10,015	15,913
18-Jun-20	4,337	10,159	16,209
19-Jun-20	4,177	10,288	16,450
20-Jun-20	4,022	10,402	16,643
21-Jun-20	3,872	10,503	16,795
22-Jun-20	3,729	10,593	16,912
23-Jun-20	3,592	10,672	17,000
24-Jun-20	3,461	10,742	17,066
25-Jun-20	3,336	10,803	17,112
26-Jun-20	3,218	10,856	17,143
27-Jun-20	3,105	10,903	17,163
28-Jun-20	2,998	10,944	17,172
29-Jun-20	2,896	10,980	17,175
30-Jun-20	2,800	11,012	17,171
1-Jul-20	2,709	11,040	17,164
2-Jul-20	2,623	11,064	17,153
3-Jul-20	2,542	11,085	17,140
4-Jul-20	2,465	11,103	17,125
5-Jul-20	2,393	11,120	17,109
6-Jul-20	2,324	11,134	17,093
7-Jul-20	2,260	11,147	17,076
8-Jul-20	2,199	11,158	17,059
9-Jul-20	2,142	11,168	17,043
10-Jul-20	2,088	11,176	17,026
11-Jul-20	2,038	11,184	17,010
12-Jul-20	1,990	11,191	16,995
13-Jul-20	1,945	11,197	16,980
14-Jul-20	1,903	11,202	16,966
15-Jul-20	1,863	11,207	16,952
16-Jul-20	1,826	11,211	16,939
17-Jul-20	1,791	11,215	16,926
18-Jul-20	1,758	11,219	16,915
19-Jul-20	1,727	11,222	16,903

**Table 5:** Predicted value Ypred for D1 using hybrid models.

**Date**	**PNN**	**GRNN**	**RBFNN**
31-May-20	5,404	5,434	5,574
1-Jun-20	5,608	5,731	6,111
2-Jun-20	5,781	6,048	6,403
3-Jun-20	5,906	6,333	6,744
4-Jun-20	5,993	6,648	7,500
5-Jun-20	6,029	6,970	8,342
6-Jun-20	6,025	7,291	8,861
7-Jun-20	5,976	7,609	9,441
8-Jun-20	5,898	7,920	10,396
9-Jun-20	5,787	8,220	11,382
10-Jun-20	5,658	8,506	11,975
11-Jun-20	5,509	8,777	12,548
12-Jun-20	5,351	9,029	13,419
13-Jun-20	5,183	9,264	14,252
14-Jun-20	5,014	9,479	14,634
15-Jun-20	4,841	9,676	14,955
16-Jun-20	4,671	9,854	15,552
17-Jun-20	4,502	10,015	16,104
18-Jun-20	4,338	10,160	16,212
19-Jun-20	4,177	10,288	16,277
20-Jun-20	4,022	10,403	16,640
21-Jun-20	3,873	10,504	16,986
22-Jun-20	3,730	10,594	16,915
23-Jun-20	3,592	10,673	16,827
24-Jun-20	3,462	10,742	17,063
25-Jun-20	3,336	10,804	17,303
26-Jun-20	3,218	10,857	17,146
27-Jun-20	3,105	10,904	16,989
28-Jun-20	2,998	10,945	17,170
29-Jun-20	2,896	10,981	17,366
30-Jun-20	2,800	11,013	17,174
1-Jul-20	2,709	11,040	16,990
2-Jul-20	2,623	11,065	17,150
3-Jul-20	2,542	11,086	17,331
4-Jul-20	2,465	11,104	17,128
5-Jul-20	2,393	11,121	16,936
6-Jul-20	2,325	11,135	17,090
7-Jul-20	2,260	11,148	17,267
8-Jul-20	2,200	11,159	17,062
9-Jul-20	2,142	11,169	16,869
10-Jul-20	2,089	11,177	17,024
11-Jul-20	2,038	11,185	17,201
12-Jul-20	1,990	11,192	16,998
13-Jul-20	1,945	11,198	16,807
14-Jul-20	1,903	11,203	16,963
15-Jul-20	1,863	11,208	17,143
16-Jul-20	1,826	11,212	16,942
17-Jul-20	1,791	11,216	16,753
18-Jul-20	1,758	11,220	16,912
19-Jul-20	1,727	11,223	17,094

Table 6 shows the predicted number of COVID-19 death cases using time series data (i.e., D2) for three standard and hybrid SNN models.

**Table 6:** Predicted death cases for D2.

**Number of confirmed cases**	**Models**		
	**PNN**	**GRNN**	**RBFNN**
200,000	5465.563	4839.117	6206.483
210,000	5469.28	4300.973	6872.048
220,000	5377.04	3797.288	7468.859
230,000	5211.424	3437.197	7938.901
240,000	4996.182	3216.437	8276.483
250,000	4751.402	3092.094	8504.179
260,000	4492.206	3025.066	8651.558
270,000	4229.201	2989.672	8744.469
280,000	3969.475	2971.118	8802.077
290,000	3717.585	2961.382	8837.423
300,000	3476.337	2956.237	8858.968

Table 7 shows the calculated MRP for COVID- 19 predicted death cases using the dataset (D1). MRP is defined as in Equation (9). It is described as the number of predicted death cases divided by the number of confirmed cases and then multiplied by 100. It shows the number of COVID-19 deaths per 100 COVID-19 confirmed cases.

(8) $$\begin{array}{r} MRP=\frac{Number\;of\;Predicted\;Death\;Cases}{Number\;of\;Confirmed\;Cases}\;*\;100 \end{array}$$

**Table 7:** MRP for D2.

**Confirmed cases**	**Models**		
	**PNN**	**GRNN**	**RBFNN**
200,000	2.7	2.4	3.1
210,000	2.6	2.0	3.3
220,000	2.4	1.7	3.4
230,000	2.2	1.5	3.4
240,000	2.1	1.3	3.4
250,000	1.9	1.2	3.4
260,000	1.7	1.2	3.3
270,000	1.6	1.1	3.2
280,000	1.4	1.1	3.1
290,000	1.3	1.0	3.0
300,000	1.1	1.0	2.9

**Figure 6:**
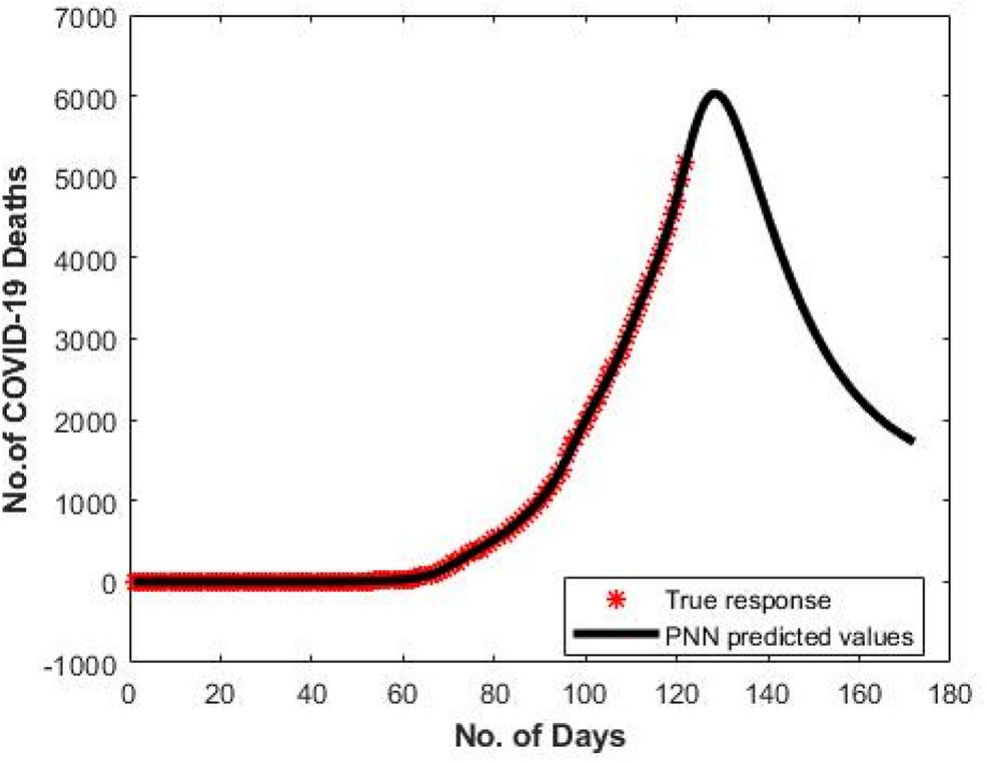
Predicted curve for D1 using PNN.

Figure 6 shows the predicted curve of PNN for COVID-19 death cases vs. the number of days since the first COVID-19 case for India, respectively. Here, the X-axis represents the number of days and Y-axis represents the number of death cases predicted. For the dataset (D1), the PNN model shows a gradual decrease in the number of death cases after the 130th day since 1st COVID-19 in India.

**Figure 7:**
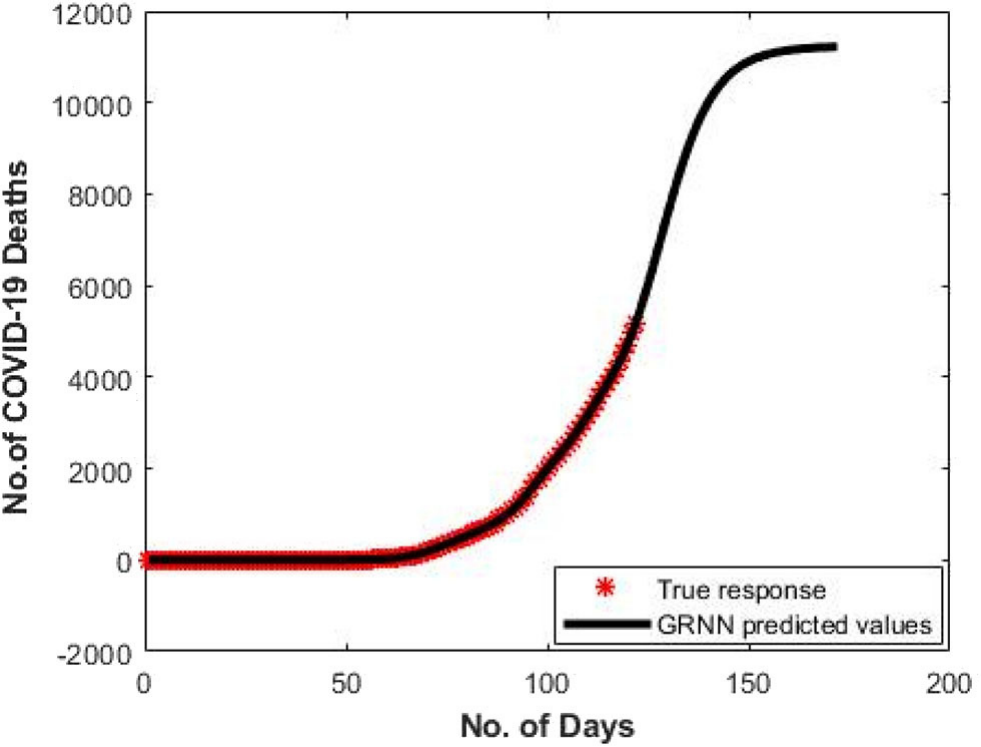
Predicted curve for D1 using GRNN.

Figure 7 denotes the predicted curve of GRNN for COVID-19 death cases vs. the number of days since the first COVID-19 case for India, respectively. Here, the X-axis signifies the number of days and Y-axis signifies the number of death cases predicted. For the dataset (D1), the GRNN model shows a smoothing means curve in the number of death cases after the 130th day since 1st COVID-19 in India, while the GRNN models show a smoothly increasing pattern.

**Figure 8:**
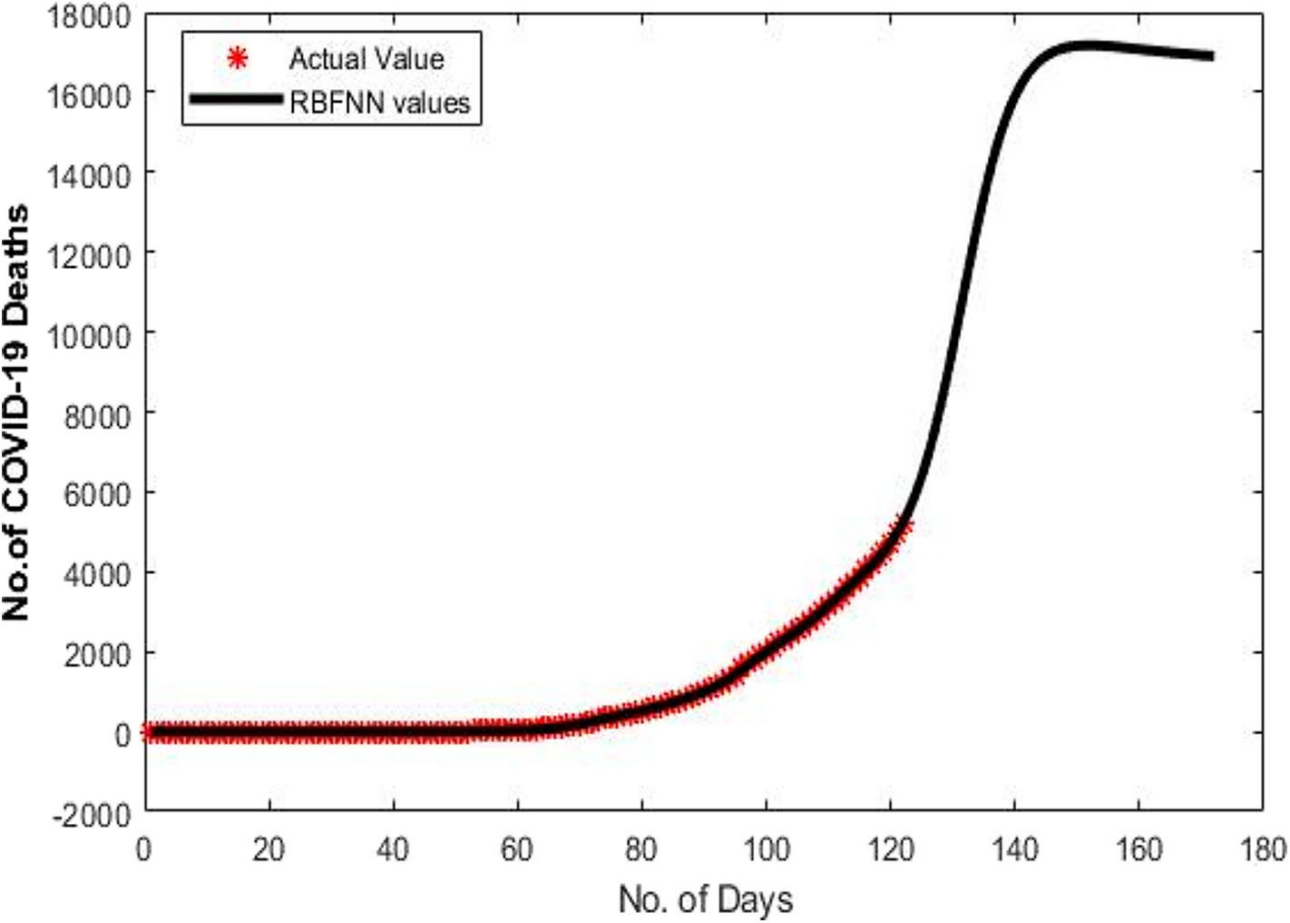
Predicted curve for D1 using RBFNN.

Figure 8 shows the predicted curve of RBFNN for COVID-19 death cases vs. the number of days since the first COVID-19 case for India, respectively. Here, the X-axis represents the number of days and Y-axis represents the number of death cases predicted. The shape of the curve is bell curve. For the dataset (D1), the RBFNN model shows a increasing in the number of death cases after the 130th day since 1st COVID-19 in India.

**Figure 9:**
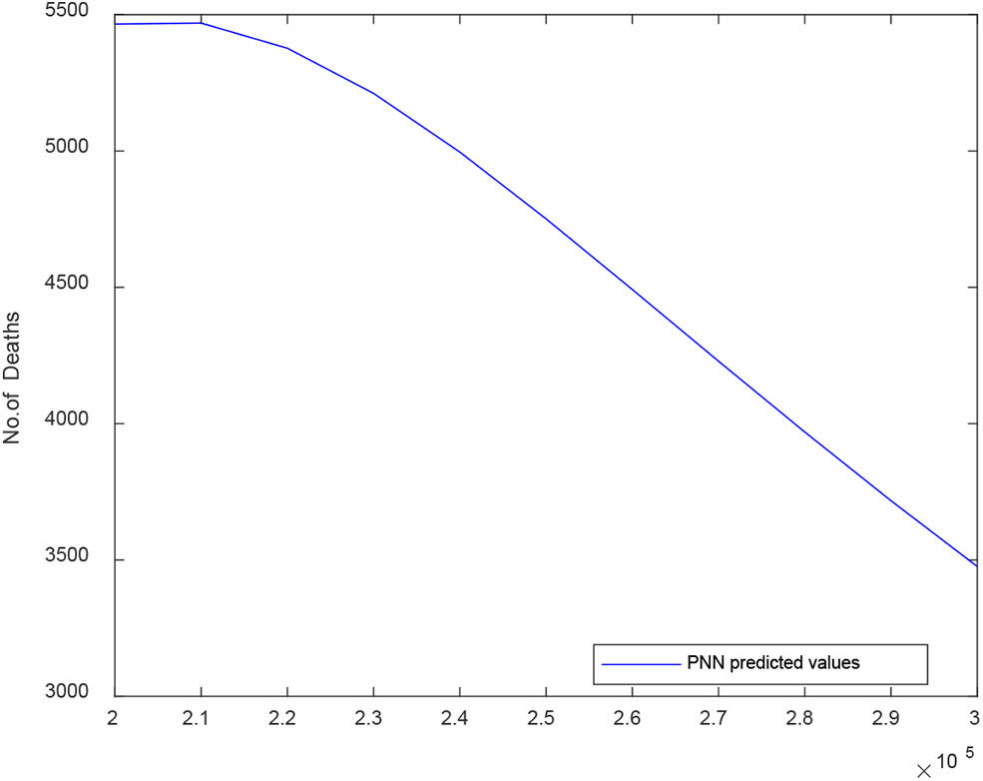
Predicted curve for D2 using PNN.

Figure 9 illustrates the predicted curve of PNN for D2 death cases vs. the number of confirmed cases for India. Here, the X-axis signifies the number of confirmed cases and Y-axis signifies the number of death cases predicted. The PNN model shows a sharp decrease in the number of death cases after the number of COVID-19 confirmed cases reach 220,000 nearly.

**Figure 10:**
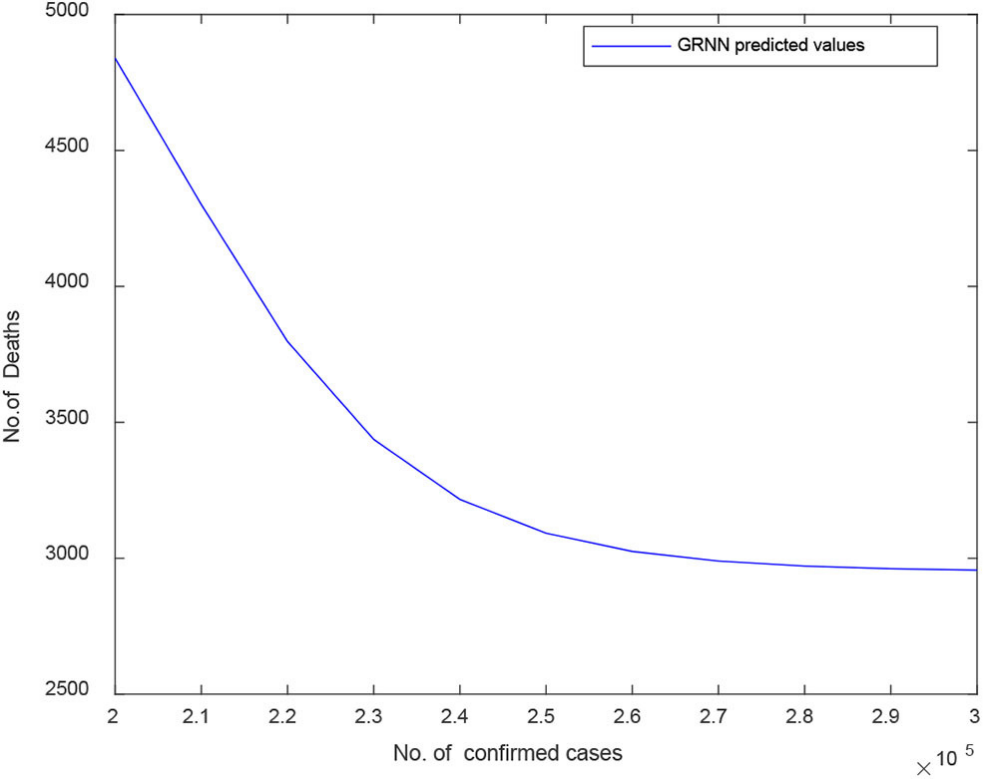
Predicted curve for D2 using GRNN.

Figure 10 proves the predicted curve of GRNN for COVID-19 death cases vs. the number of confirmed cases for India. Here, the X-axis depicts the number of confirmed cases and Y-axis signifies the number of death cases predicted. The GRNN model shows a decrease pattern in the number of death cases after 245,000 COVID-19 confirmed cases.

**Figure 11:**
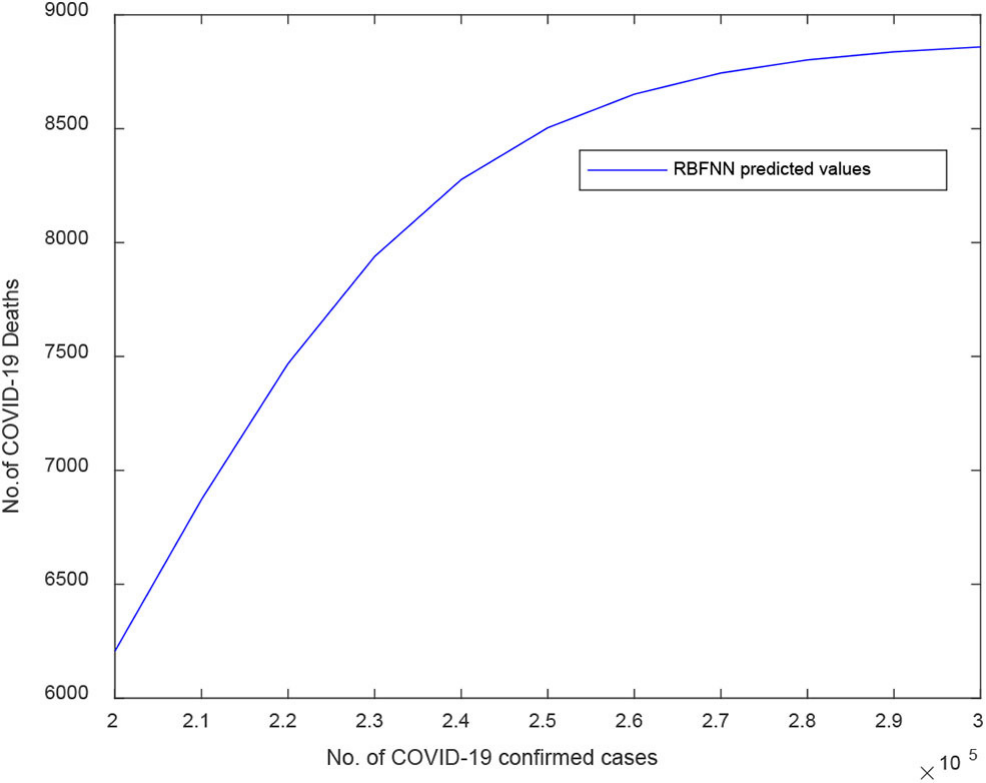
Predicted curve for D2 using RBFNN.

Figure 11 illustrates the RBFNN predicted curve for COVID-19 death cases vs. the number of confirmed cases for India. Here, the X-axis signifies the number of confirmed cases and Y-axis signifies the number of death cases predicted. The RBFNN shows an increasing pattern in the number of death cases after 245,000 COVID-19 confirmed cases.

**Figure 12:**
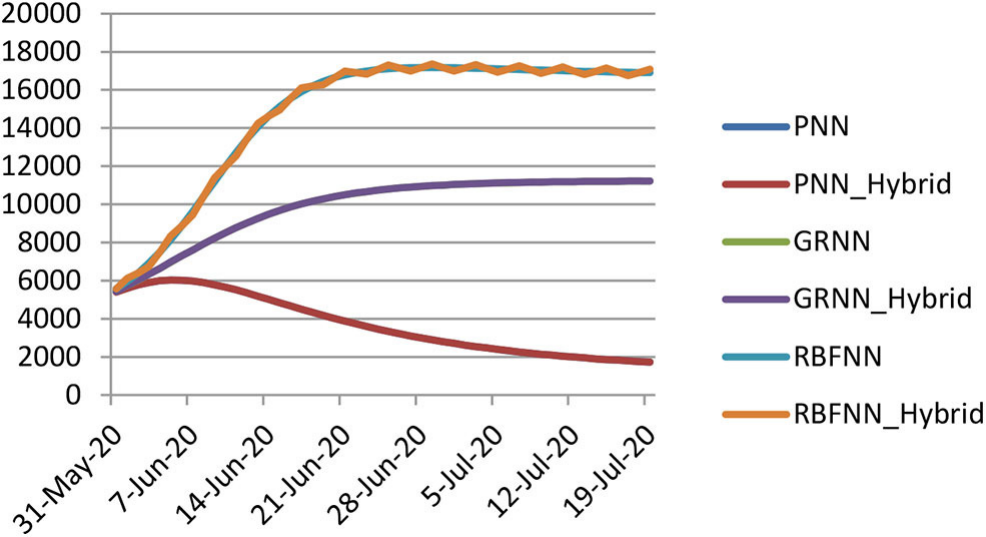
Comparison of SNN models and hybrid models for D1.

Figure 12 depicts that forecasted error is very less or almost zero in the case of PNN and GRNN whereas RBFNN model shows slightly high error value.

The advantage of this study is that the COVID-19 mortality rate prediction using the SNN model and its hybrid models and gives a profound and solid comprehension of the pattern and qualities of COVID-19. An important observation was made from this investigative performance study of SNN models for the COVID-19 datasets, that no single neural network model can be considered the best model, which depends entirely on the neural network parameters and the characteristics of the data.

### Limitations

There is some limitation of our current work, which are as follows:

- First, COVID-19 is initially recognized as mild illness with dry cough, in more cases there are asymptomatic and seldom leads to death. The majority of COVID-19 cases in India is asymptomatic and very mildly infected individuals, which they are not available to human services experts, which resulted in under-reporting.
- Second, other demographical and topographical components related with the event and spread of COVID-19 are excluded from the proposed SNN models; thus, regardless of whether the SNN models consider these factors, encourages the improvement in the prescient exactness will require further confirmation.
- Lastly, the hybrid SNN-NAR-NN model is developed based on the benchmark neural network regression model that is suited for short-term mortality rate prediction very well. Finally, applicability of these SNN models in other infectious diseases may be carried out as future work.

## Conclusion and Future Work

This research paper proposed a SNN models and their hybrid version with the NAR-NN time series model for the prediction of the COVID-19 mortality rate in India. The performances of these models have evaluated by using RMSE and “R,” a correlation value. Based on the comparison of the RMSE values of these models, it was found that the SNN models for D1 are higher than D2. Therefore, in this work, SNN is hybridized with NAR-NN for dataset D1 to predict the future error of the SNN models, which was added to the predicted value of these models for better mortality rate prediction. On the whole, the empirical results were showed that: (i) RBFNN based MRP model performed better than the GRNN and PNN models for D1 dataset, (ii) PNN based MRP model performed better than the GRNN and RBFNN models for D2 dataset. For the both datasets, SNN based MRP models have captured the incremental curve for COVID-19 death cases for India. The proposed method is capable of providing a predictive tool for assessing its current state of infection, severity, and help government and health care workers for better decision making to reduce the mortality rate in India.

In the future, deep learning Recurrent Neural Network time series forecasting model will be used to increase the prediction accuracy for the COVID-19 mortality rate prediction. And also, this study will be enhanced by including many factors or variables like demographical factors, geographical factors, and weather factors (temperature, humidity, wind speed, and rainfall) for modeling the highly accurate prediction model for ongoing COVID-19 pandemic.

## Data Availability Statement

Publicly available datasets were analyzed in this study. This data can be found here: https://www.kaggle.com/sudalairajkumar/novel-corona-virus-2019-dataset?select=time_series_covid_19_deaths.csv.

## Author Contributions

SD and RR: conceptualization, implementation, and design. SD, RR, and JC: writing and editing. JC: verification. All authors contributed to the article and approved the submitted version.

## Conflict of Interest

The authors declare that the research was conducted in the absence of any commercial or financial relationships that could be construed as a potential conflict of interest.
